# The complete mitochondrial genome of the lesser sac-winged bat *Saccopteryx leptura* (Chiroptera: Emballonuridae) from Costa Rica

**DOI:** 10.1080/23802359.2017.1318679

**Published:** 2017-04-21

**Authors:** José Horacio Grau, Martina Nagy, Emrah Coraman, Frieder Mayer

**Affiliations:** a Museum für Naturkunde Berlin, Leibniz-Institut für Evolutions- und Biodiversitätsforschung, Berlin, Germany;; b Animal Behavior Lab, Free University Berlin, Berlin, Germany;; c Institute of Environmental Sciences, Bogazici University, Bebek, Istanbul, Turkey

**Keywords:** *Saccopteryx leptura*, lesser sac-winged bat, Emballonuridae, Chiroptera, mitogenome

## Abstract

Here we present the first complete mitochondrial genome of the lesser sac-winged bat *Saccopteryx leptura* (Chiroptera: Emballonuridae) from Costa Rica, assembled from next-generation sequencing data. The mitogenome of *Saccopteryx leptura* measures 16,577 bp in length, and contains 13 protein-coding genes, 2 ribosomal RNA genes and 22 transfer RNA genes. A slight A + T bias was observed in the mitogenome of *Saccopteryx leptura* with an overall base composition of 31.5% A, 28.3% T, 25.8% C, and 14.2% G, and a GC content of 40.1%. The gene arrangement was identical to that of previously described bat mitogenomes.

The family of Emballonuridae comprises 51 bat species and is found in the old as well as in the new world (Simmons 2005). The Neotropical genera and especially the genus *Saccopteryx* have recently received much attention since several species show complex social behaviour including unusual mammalian sex-biased dispersal (Nagy et al. 2007) and vocal learning (Knörnschild 2014). Finally, high genetic divergence in the mitochondrial genome was found throughout the species’ distribution ranges indicating the existence of cryptic species diversity (Clare et al. 2011). Reliable phylogenetic and population genetic analyses involving also the mitochondrial genome will help to understand the divergent evolution of behavioural traits within the Neotropical emballonurids. Here we present the full mitogenome of the Neotropical bat species *Saccopteryx leptura* (Schreber 1774).

An adult male was caught in Costa Rica at La Selva Biological Station (10.4311°N, 84.0052°W) on 21 August 2008 (research permit 183-2008-SINAC). A small wing tissue biopsy was taken prior to the release of the bat.

Genomic DNA was isolated according to a salt-chloroform procedure (modified from Müllenbach et al. 1989). Five hundred nanograms were used for fragmentation with Covaris Ultrasonificator (AFA Technology, Woburn, MA). Hundred nanograms of sheared DNA were used for Illumina Library Preparation with NEXTflex Rapid DNASeq Kit (Biooscientific, Austin, TX). 150 bp Paired End Sequencing was performed with Illumina´s HiSeq 2500 Sequencer (Rapid Mode, San Diego, CA) using one third of a flowcell. Shotgun sequencing yielded a total of 70,558,564 paired end reads. A complete circularized mitochondrial genome was obtained with NOVOplasty 2.4 (Dierckxsens et al. 2017) using kmer 47. Annotations were carried out with MITOchondrial genome annotation Server (MITOS) (Bernt et al. 2013), and manual validation of the coding regions was done using the NCBI ORF Finder (http://www.ncbi.nlm.nih.gov/gorf/gorf.html).

The annotated sequence file was submitted to NCBI (accession no. KY681816). The phylogenetic position of the new sequence of *S. leptura* based on the cytochrome B gene is shown in Figure 1.

**Figure 1.**
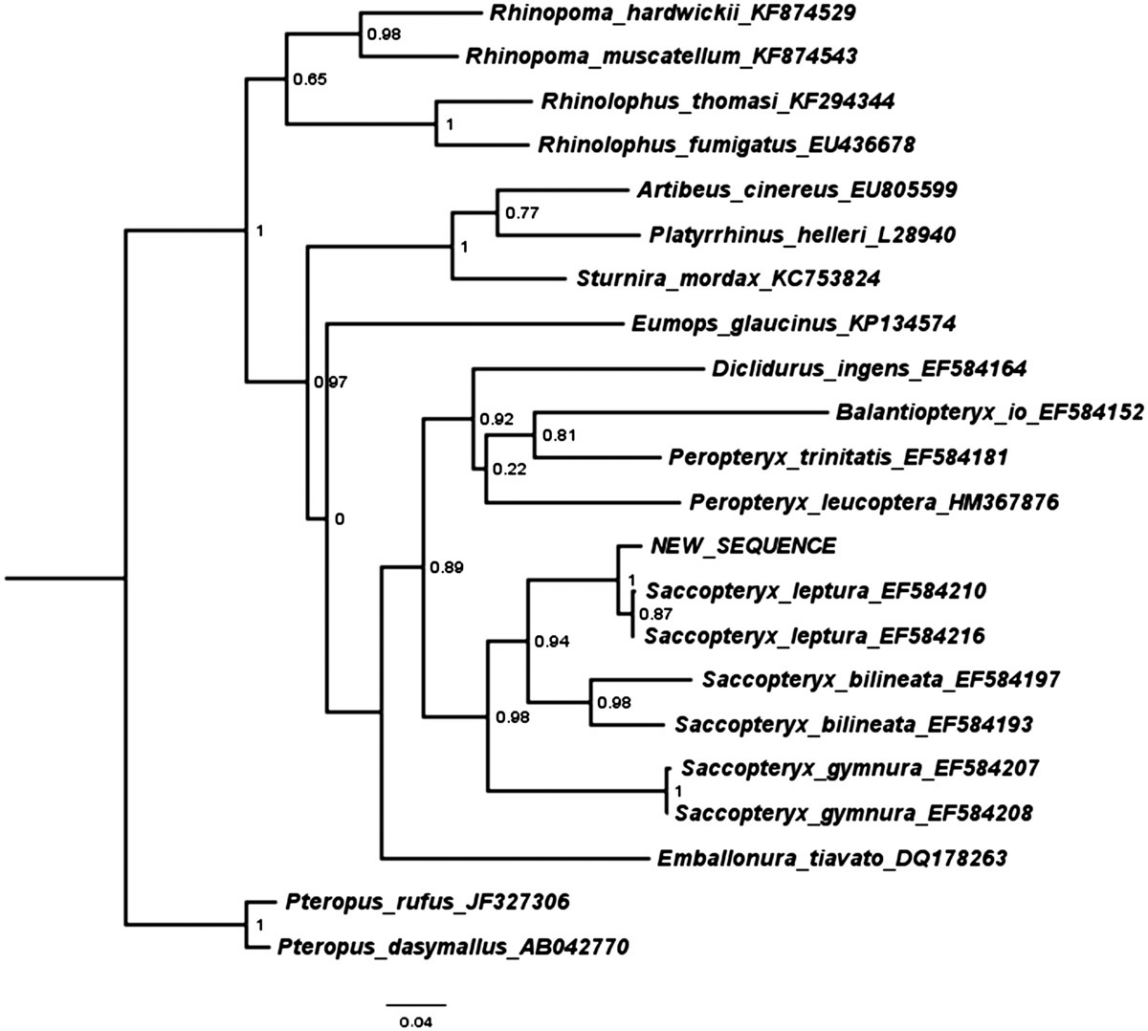
Maximum likelihood tree illustrating the phylogenetic position of the newly sequenced *Saccopteryx leptura* gene sequence among a subset of bat species. Cytochrome B sequences were aligned using MAFFT 7.271 and highly divergent or poorly aligned regions were removed with Gblocks 0.91b (Castresana 2000) allowing for gap positions and smaller blocks. Trees were calculated using PhyML 3.1 (Guindon et al. 2010) with four rate categories, optimized equilibrium frequencies, GTR model of sequence evolution and combined heuristics (Nearest Neighbour Interchange and Subtree Pruning and Rerafting).

The complete mitochondrial transcript of *Saccopteryx leptura* was 16,577 bp in length and contained 13 protein-coding genes (PCGs), 2 ribosomal RNA genes and 22 transfer RNA genes. As described for other bat mitogenomes (Yoon & Park 2016; Yoon et al. 2016), the mitochondrial genome of *Saccopteryx leptura* contained a slight A + T bias with an overall base composition of 31.5% A, 28.3% T, 25.8% C, and 14.2% G, and a GC content of 40.1%. The gene arrangement of the present mitogenome is similar to that of other bats (Yu et al. 2016; Jiang et al. 2016). Most of the genes were encoded on the L-strand except for *ND6* and eight tRNA genes (*tRNA^Gln^, tRNA^Ala^, tRNA^Asn^, tRNA^Cys^, tRNA^Tyr^, tRNA^Ser2^, tRNA^Glu^* and *tRNA^Pr^°*), which were encoded in the H-strand. All PCGs had ATG as initiation codon with the exception of *ND3 and ND5* which used ATA as initiation codon. TAA was the most used termination codon except for *CYTB*, which used an AGA termination codon. Incomplete stop codons (T– or TA–) were found in *COX3* and *ND4*. The *12S* and *16S* genes had a length of 967 and 1559 bp, respectively.
